# Validity and Reliability of the Track-UL Algorithm Compared With Kinovea Software for Measuring Upper-Limb Functional Range of Motion in People After Stroke: Cross-Sectional Observational Study

**DOI:** 10.2196/87128

**Published:** 2026-05-11

**Authors:** Hatem Lazem, David Harris, Abi Hall, Thomas C Richards, Phaedra Leveridge, Maedeh Mansoubi, Xiaohan Xu, Paul Newell, Sarah E Lamb, Helen Dawes

**Affiliations:** 1 Medical School, Faculty of Health and Life Sciences, University of Exeter, St Luke's Campus, Exeter, EX1 2LP, United Kingdom, +44 7436 959585; 2 Basic Science Department, Faculty of Physical Therapy, Cairo University, Giza, Egypt; 3 Faculty of Health and Life Sciences, NIHR Exeter Biomedical Research Centre, University of Exeter, Exeter, United Kingdom; 4 School of Public Health and Sport Science, Faculty of Health and Life Sciences, University of Exeter, Exeter, United Kingdom

**Keywords:** stroke, telemonitoring, range of motion, MediaPipe, markerless tracking, validity, reliability

## Abstract

**Background:**

Approximately 70% of survivors of stroke have problems with arm function. Physiotherapists assess arm functional range of motion (ROM) using either a goniometer or functional questionnaires, which lack objective accuracy and require a skilled physiotherapist. We developed the Track-UL algorithm based on a markerless motion capture system to measure arm ROM.

**Objective:**

This study aimed to measure the agreement between our novel Track-UL algorithm and Kinovea software in assessing arm ROM during functional tasks in the laboratory and home settings.

**Methods:**

Videos were recorded while 27 survivors of chronic stroke performed 4 functional tasks (forward reaching, arm abduction, moving the hand toward the mouth, and moving the hand toward the head) in the laboratory and at home. The videos were analyzed by 2 independent raters using the Track-UL algorithm and Kinovea software. The limits of agreement and intraclass correlation coefficients were calculated.

**Results:**

We found no clinically significant systematic bias in shoulder and elbow angle, with good agreement between the Track-UL algorithm and Kinovea software (assessed via Bland-Altman plots). The 95% limits of agreement were –3.18 to 6.41 degrees for the shoulder joint and −5.35 to 8.78 degrees for the elbow joint in the laboratory setting, and –6.21 to 3.62 degrees for the shoulder joint and −4.06 to 2.53 degrees for the elbow joint in the home setting. There was excellent absolute agreement between the measurement tools across all tasks and joints; intraclass correlation coefficient values ranged from 0.97 (95% CI 0.97-0.99) to 0.99 (95% CI 0.99-0.99; *P*<.001 for both laboratory and home measurements).

**Conclusions:**

The novel Track-UL algorithm is an accurate, valid, and easy tool that can be used to assess upper-limb ROM in survivors of stroke at clinics and potentially at home. This will support physiotherapists in remotely monitoring and adapting rehabilitation programs.

## Introduction

Stroke is the second leading cause of death and the third leading cause of long-term disability for approximately 70% of people who experience a stroke, affecting their quality of life [1]. Approximately 40% of people with stroke experience upper-limb problems at the chronic stage of recovery [2]. Following a stroke, people can experience muscle weakness [3], spasticity [4], and changes in the pattern of arm mobility on the affected side [5].

According to the National Institute for Health and Care Excellence guidelines, physiotherapists routinely design rehabilitation programs to improve upper-limb function and, therefore, need to efficiently and effectively assess and monitor improvements in the functional range of motion (ROM) in people with stroke [6]. In clinical settings, physiotherapists typically use a clinical goniometer [7] or validated clinical questionnaires such as the Fugl-Meyer Assessment for the upper extremity (FMA-UE) [8] to evaluate and monitor the upper-limb ROM in people with stroke.

A goniometer is the current standard tool for assessing ROM in physiotherapy practice [9]. However, this requires an adequate level of expertise from the practitioner to execute, so its precision can be influenced by clinical skills and experience [10,11]. In addition, the presence of compensation during upper-limb movements, such as shoulder and trunk rotation, and the disruption of elbow-shoulder coordination can affect the ease with which goniometers can be used to measure ROM [12]. A limitation of upper-limb clinical assessment questionnaires such as the FMA-UE scale is the time required to complete them, which can be up to 25 minutes. They also rely on a Likert-based rating system in which scores of 0, 1, and 2 indicate no movement, partial movement, and full movement, respectively, which is discrete and bounded, depends on the raters’ experience, and lacks the precision in quantifying progression and monitoring small changes in arm mobility over time [8].

An alternative approach is the use of affordable and free 2D markerless motion analysis tools such as the Kinovea software that allow therapists to measure the upper-limb kinematics from video footage [13]. However, such tools are better suited to research as the time and expertise of an experienced health care professional required for operation make them less accessible to physiotherapists in clinics. Computer vision algorithms based on markerless motion capture and pose estimation frameworks offer a potential solution for faster and easier measurement of the ROM without the need for an experienced health care professional [14]. So far, computer vision–based algorithms have been validated for people with ankylosing spondylitis [15] and following knee replacement [16] but are still in need of validation among people with stroke, who have different movement complexities such as compensatory movement patterns and reduced ROM, which differ from the normative datasets on which most algorithms are trained, which can introduce the possibility of tracking errors [12,17,18].

Therefore, based on the gap in the current literature on the validity and reliability of the pose estimation algorithms in stroke, the aim of this study was to assess the concurrent validity and test-retest reliability of a novel Track-UL algorithm based on MediaPipe, a pose estimation framework, as an objective and easy-to-use tool for measuring and monitoring upper-limb mobility in people with stroke in the laboratory and home settings.

The main objectives of this study were as follows:

- To investigate the concurrent validity of the Track-UL algorithm based on a 2D pose estimation framework compared to the Kinovea software for measuring shoulder and elbow joint functional active ROM
- To investigate the test-retest reliability of the Track-UL algorithm based on a 2D pose estimation framework for measuring shoulder and elbow joint functional active ROM
- To explore the potential for using the Track-UL algorithm as a telemonitoring tool for measuring upper-limb functional active ROM from videos recorded by people with stroke in their own homes

## Methods

### Ethical Considerations

This study was conducted in accordance with the ethical standards of the responsible institutional and national committees on human experimentation and with the World Medical Association Declaration of Helsinki (1975, as revised in 2013). This project was reviewed and approved by an independent National Health Service research ethics committee and the Health Research Authority (reference 24/LO/0434). Written informed consent was obtained from all participants prior to their inclusion in the study. The privacy and confidentiality of participants’ data and identities were strictly maintained. Personally identifiable information was stored securely and separately from research data, with access limited to authorized members of the research team. Participants received compensation for travel expenses associated with study participation.

### Participants

This was a cross-sectional observational study in which a total of 27 people with stroke participated. Data collection was conducted in the laboratory by the researcher (a physiotherapist) at the University of Exeter and participants’ homes. We recruited people with stroke aged 18 years or older who were more than 6 months after stroke with a score of 2 or lower on the Modified Ashworth Scale and any degree of upper-limb impairment resulting from stroke (FMA-UE score<57). Survivors of stroke were excluded if they had severe cognitive deficits and could not follow the instructions given by the researcher or had severe spasticity (score of >2 on the Modified Ashworth Scale). The reason for these exclusions was that these patient groups are not able to achieve active movements, which are a requirement of the tasks this study aimed to validate.

### Procedure

**Figure 1.**
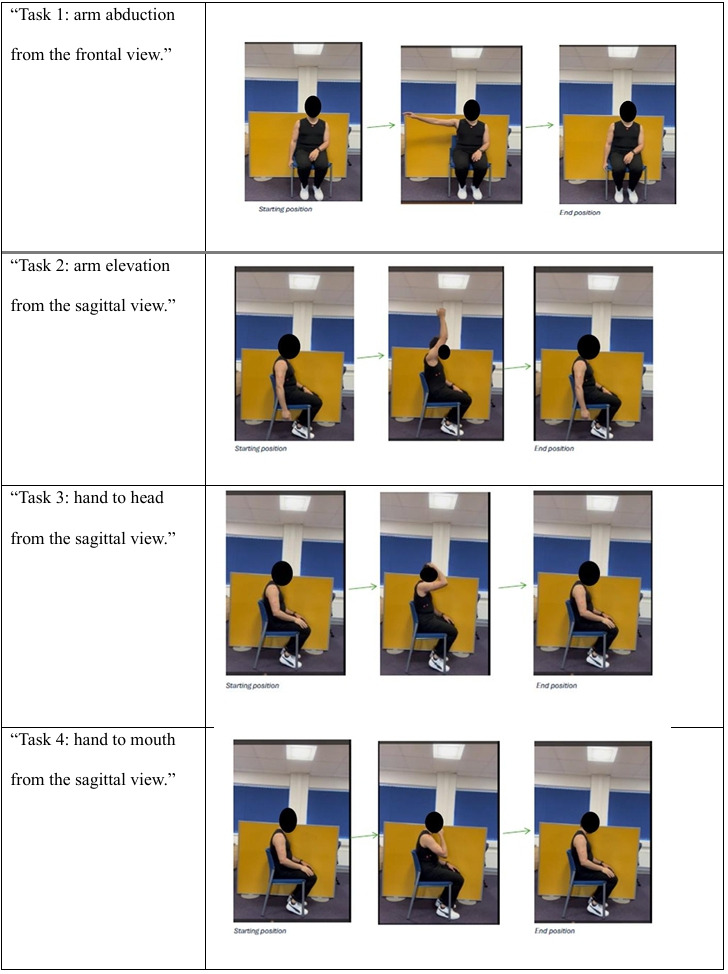
The 4 functional complex upper-limb tasks.

All participants completed a single face-to-face laboratory testing session at the University of Exeter with a physiotherapist, which lasted up to 60 minutes, including collection of demographic data and assessment of upper-limb ROM, with videos recorded while participants performed 4 functional upper-limb tasks (Figure 1). Participants were then asked to repeat the same 4 tasks alone at home with or without support from their carers following written instructions provided by the research team while recording videos of the tasks within 3 days following the initial assessment.

To familiarize participants with the tasks, each movement was demonstrated to them, who then practiced it twice before starting. Participants were asked to wear light clothes with sleeveless shirts to aid identification of bony landmarks on the upper limb. Before recording the videos, colored circular stickers (markers) were applied to predefined bony landmarks of the upper limb (acromion process, anterior and midthoracic line, lateral epicondyle, midshaft of the humerus, midforearm, and midwrist). The use of these markers enabled measurement of shoulder and elbow range of movements during the 4 tasks (Figure 1). For the videos recorded at home by the participants or their carers, we used virtual markers implemented in the Kinovea software.

All videos were imported into a laptop and analyzed using both the Kinovea software and PyCharm Community Edition (version 2022.3.1; JetBrains) using the Track-UL algorithm, a function programmed in PyCharm. This function calculates the angle of joints of the human body based on the joint coordinates using the 2D vector dot product formula. All videos were recorded by 1 physiotherapist. Two physiotherapists (one of whom recorded the videos) who were blinded to each other’s measurements then independently analyzed the video recordings: one using the Kinovea software and the other using the Track-UL algorithm.

For home testing, we asked the participants themselves or their carers to record the videos only for the affected side within 3 days of the laboratory test and then send the videos back to the researcher.

### Instruments

Kinovea is free and open-source analysis software used by health care professionals in research and clinical settings. It is considered to be a valid and reliable tool for measuring ROM using a digital goniometer, which is widely used in clinical and sports biomechanics for 2D kinematic analysis [19,20]. While the gold standard for measuring ROM is the 3D motion capture system, using Kinovea allows for a more applicable comparison aligned with the intended use of this algorithm in real-world environments such as a clinic or the home setting, in which stroke assessment and telerehabilitation usually occur. Analysis of the captured videos was carried out using Kinovea (version 0.9.5; Joan Charmant). Circular colored markers were placed on the bony landmarks of the upper limb prior to capture to facilitate calculation of upper-limb joint angles using a digital goniometer.

**Figure 2.**
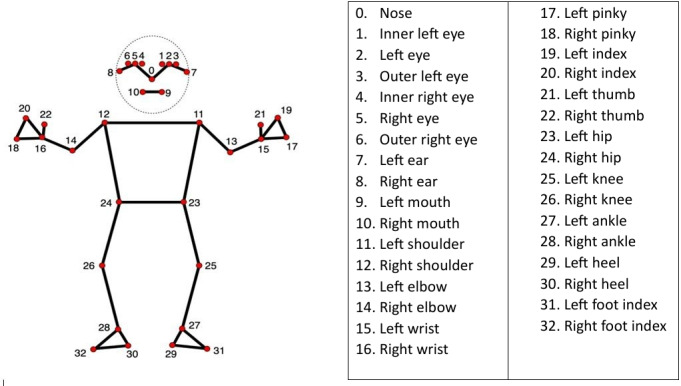
BlazePose model of 33 human poses [22].

MediaPipe is an open-source pose estimation model developed by Google for machine learning applications [21] (Figure 2 [22]). The model infers 33 pose landmarks from the whole body; offers real-time processing and compatibility with standard video input from commonly available devices such as mobile phones; and offers a practical balance between accuracy and usability [23] compared to other pose estimation software such as OpenPose [24] and OpenCap [25] that require high computational resources and more complex setup, which can limit their applicability in a clinical setting or at home. The Track-UL algorithm was developed using PyCharm for automatic calculation of both shoulder and elbow joint angles from videos, which are then presented to the user. To calculate joint angles, coordinates for bony landmarks (hip, shoulder, elbow, and wrist) were extracted from video frames using the MediaPipe pose estimation framework. Each landmark provides 2D coordinates (*x*, *y*). Shoulder and elbow joint angles were computed by treating the connected rigid body segments (trunk, upper arm, and forearm) as 2D vectors (Figures2  3 [22]).

**Figure 3.**
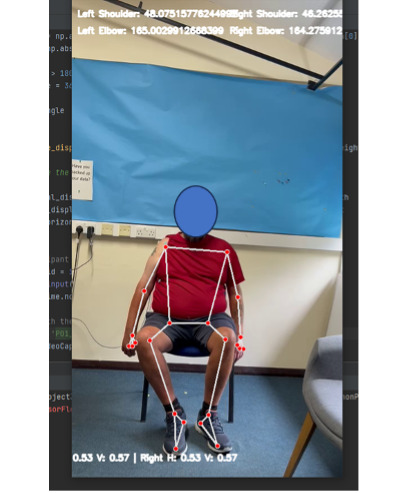
How the video appears to the physiotherapist when we apply the Track-UL algorithm.

A 2D vector dot product formula was used to calculate shoulder joint angle (term B in the following formulas) using coordinates of the shoulder joint and the adjacent hip (term A) and elbow (term C) joints:


$$\theta \text{=}arccos\left(\frac{\left(A\text{-}B\right)\cdot \left(C\text{-}B\right)}{\left\|A\text{-}B\right\|\left\|C\text{-}B\right\|}\right)$$

$$\begin{array}{c} \theta =arrco\;s(((xA-xB)\cdot (xC-xB)+(yA-yB)\cdot (yC-yB))/\\ \left(\sqrt{\left((xA-xB{)}^{2}+(yA-yB{)}^{2}\right)}\times \sqrt{((xC-xB{)}^{2}+(yC-yB{)}^{2}}))\right) \end{array}$$

### Video Processing

The researcher recorded 8 videos of 4 different upper-limb functional tasks performed on each side (affected and unaffected side) using a mobile phone camera (iPhone 13 Pro Max [Apple Inc] with a 12-MP main camera with sensor-shift stabilization, 1.9-µm pixels, and a 26-mm equivalent f/1.5-aperture lens) and a tripod. The camera was placed 1.5 m from the participants on a tripod at a height of 90 cm oriented with an angle of 90 degrees perpendicular to the chair. Participants were then asked to record the same videos at home following standardized instructions (Multimedia Appendix 1) within the following 3 days and only for the affected side to lower the risk of fatigue from repeated movements. Each task was repeated 3 times independently in each video with 10 seconds of rest in between each repetition and one 1-minute rest in between each task. During analysis, there were no apparent challenges regarding landmark detection by the Track-UL algorithm.

This study measured the functional active ROM for both the shoulder joint (flexion and abduction) and the elbow joint (flexion and extension) during 4 different functional complex tasks, which are routinely used in the clinic as part of the FMA-UE assessment. We measured both the affected and unaffected sides to understand the extent to which the Track-UL algorithm can estimate the functional active ROM accurately on the unaffected side as well as the affected side, which can include some substitute movements due to the motor impairments compared to the unaffected side.

### Analysis

We used SPSS Statistics (version 29; IBM Corp) for data analysis. To assess the Track-UL algorithm’s concurrent validity against Kinovea, absolute agreement was evaluated using Bland-Altman analysis [26] to obtain the 95% limit of agreement (LoA) and mean bias metrics using the following equation: LoA = mean difference ± 1.96 × SD difference.

To measure the absolute agreement between our algorithm and Kinovea, we calculated the intraclass correlation coefficient (ICC) based on a 2-way random-effect model for a single measurement with an absolute agreement model (ICC(2,1)). This model accounts for both participant variability and systematic differences between the 2 assessment tools.

To assess the test-retest reliability for the Track-UL algorithm, we also calculated the ICC(2,1) for the repeated measures, the SE of measurement, and the minimum detectable change using the following equations [27,28]:


$$SEM=StDev\cdot \sqrt{1-ICC}$$

$$MDC=SEM\cdot \sqrt{2\times 1.96}$$

ICC values between 0.81 and 1.0 are interpreted as very good or excellent, ICC values between 0.61 and 0.80 are interpreted as good, ICC values between 0.41 and 0.60 are interpreted as moderate, ICC values between 0.21 and 0.40 are interpreted as fair, and ICC values below 0.20 are interpreted as poor [29].

## Results

### Overview

The demographic data of the participants are listed in Table 1.

**Table 1.** Participants’ demographic data (N=27).

Characteristics	Participants
Age (years), mean (SD; range)	60.56 (13.82; 26-88)
Time since stroke (months), mean (SD; range)	42.93 (33.07; 6-126)
FMA-UE^a^ score, mean (SD; range)	32.15 (16.21; 10-55)
MAS^b^ score—shoulder, mean (SD; range)	1.22 (0.42; 1-2)
MAS score—elbow, mean (SD; range)	1.44 (0.51; 1-2)
MAS score—hand, mean (SD; range)	1.41 (0.50; 1-2)
Sex, n (%)	
Male	21 (77.8)
Female	6 (22.2)
Type of stroke, n (%)	
Ischemic	19 (70.4)
Hemorrhagic	8 (29.6)
Affected side, n (%)	
Right	16 (59.3)
Left	11 (40.7)

a FMA-UE: Fugl-Meyer Assessment for the upper extremity.

b MAS: Modified Ashworth Scale.

### Concurrent Validity for Laboratory Measurement

**Figure 4.**
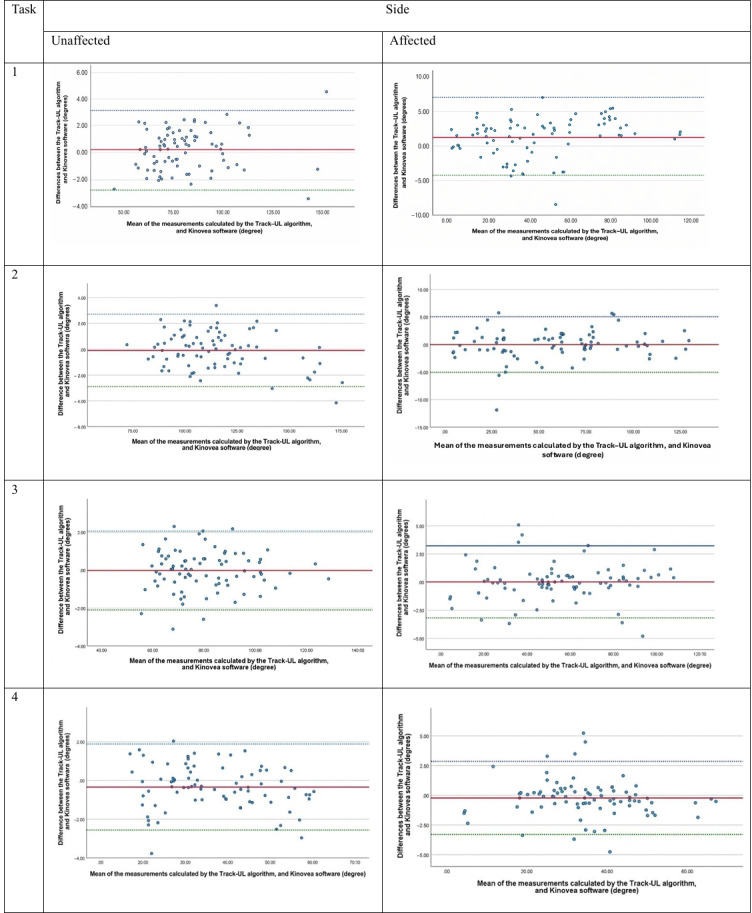
Bland-Altman plots for comparing the Track-UL algorithm and Kinovea software for the shoulder joint in both the unaffected and affected side in the laboratory setting.

The Bland-Altman plots (Figures4  5) reveal that the mean difference in shoulder and elbow joint angles between the Track-UL algorithm and Kinovea was close to 0, with a mean value ranging from −0.34 (SD 1.13) to 0.76 (SD 1.25) degrees for the unaffected side and −1.24 (SD 3.84) to 1.17 (SD 2.67) degrees for the affected side, indicating no systematic bias. The 95% LoA for joint measurements on the unaffected side ranged from −2.88 to 3.03 degrees for the shoulder and from −3.41 to 3.21 degrees for the elbow (Table 2). On the affected side, the 95% LoA ranged from −3.18 to 6.41 degrees for the shoulder and −5.35 to 6.29 degrees for the elbow (Table 2). This difference is not considered clinically significant [30]. There are a few outliers in some of the graphs; however, there is no clear evidence of a trend or heteroscedasticity in the plots except for the shoulder joint during shoulder abduction (task 1) on the affected side. In this case, a proportional bias was observed: as the abduction angle increased, the positive difference also increased. However, this positive difference of up to 6.41 degrees does not have a clinically significant impact on the measurement of the shoulder functional active ROM [30].

**Figure 5.**
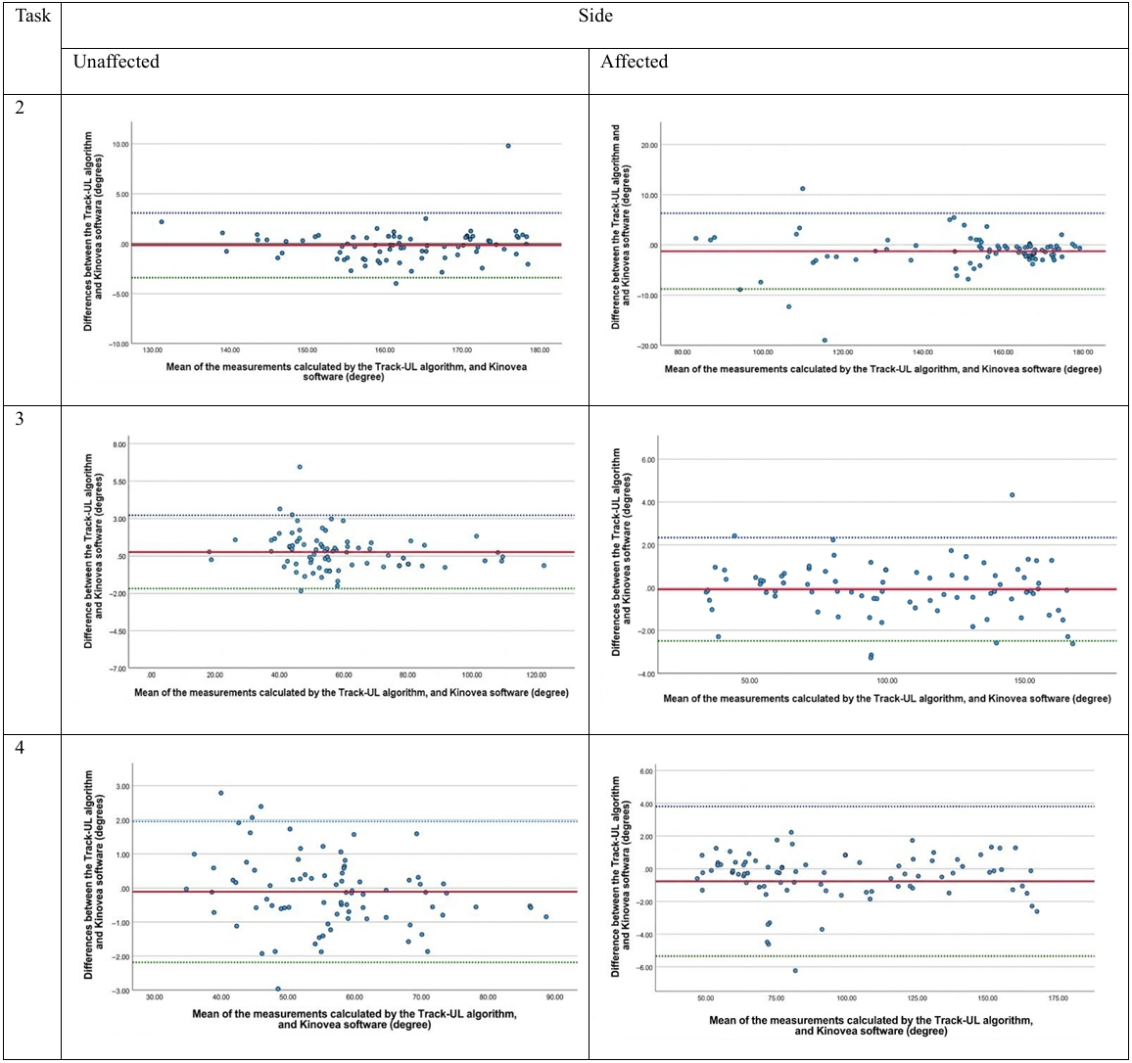
Bland-Altman plots for comparing the Track-UL algorithm and Kinovea software for the elbow joint in both the unaffected and affected side in the laboratory setting.

**Table 2.** Mean values and limit of agreement (LoA) between the Track-UL algorithm and the Kinovea software based on the laboratory measurements.

Tested side and task number	Elbow joint				Shoulder joint			
	Observations, n	Values, mean (SD; 95% CI)	Lower LoA	Upper LoA	Observations, n	Values, mean (SD; 95% CI)	Lower LoA	Upper LoA
Unaffected side								
1	—^a^	—	—	—	81	0.21 (1.45; –0.12 to 0.52)	−2.64	3.03
2	81	−0.16 (1.65; –0.52 to 0.21)	−3.41	3.08	80	−0.09 (1.43; –0.40 to 0.23)	−2.88	2.71
3	81	0.76 (1.25; 0.48 to 1.03)	−1.69	3.21	81	−0.02 (1.06; –0.26 to 0.21)	−2.10	2.06
4	81	−0.11 (1.05; –0.34 to 0.11)	−2.19	1.95	81	−0.34 (1.13; –0.59 to –0.09)	−2.56	1.87
Affected side								
1	84	—	—	—	84	1.17 (2.67; 0.59 to 1.75)	−4.06	6.41
2	84	−1.24 (3.84; –2.07 to –0.40)	−8.78	6.29	84	−0.01 (2.58; –0.57 to 0.55)	−5.07	5.05
3	84	−0.07 (1.23; –0.34 to 0.18)	−2.49	2.33	84	0.02 (1.63; –0.34 to 0.37)	−3.18	3.22
4	84	−0.77 (2.33; –1.27 to –0.26)	−5.35	3.81	84	−0.23 (1.56; –0.57 to 0.11)	−3.31	2.84

a Not applicable.

### Absolute Agreement Analysis for Laboratory Measurement

We used the ICC to assess the absolute agreement between the Track-UL algorithm and the Kinovea software when measuring ROM from videos recorded in the laboratory. The ICC(2,1) for the absolute agreement was calculated, showing excellent agreement across all tasks, joints, and sides (unaffected and affected). The ICC values ranged from 0.98 to 0.99 for the unaffected side and from 0.98 to 0.99 for the affected side (Table 3). This indicates that measurements were very consistent between the 2 assessment tools.

**Table 3.** Intraclass correlation between the Track-UL algorithm and the Kinovea software (laboratory setting).

Side and task number	Joint	Intraclass correlation coefficient (95% CI)	*F* test (*df*)	*P* value
Unaffected side				
1	Shoulder	0.99 (0.99-0.99)	714.557 (80, 80)	<.001
2	Shoulder	0.98 (0.98-0.99)	156.212 (80, 80)	<.001
2	Elbow	0.98 (0.98-0.99)	156.212 (80, 80)	<.001
3	Shoulder	0.99 (0.99-0.99)	879.210 (80, 80)	<.001
3	Elbow	0.99 (0.99-0.99)	1027.538 (80, 80)	<.001
4	Shoulder	0.99 (0.99-0.99)	473.902 (80, 80)	<.001
4	Elbow	0.99 (0.99-0.99)	481.433 (80, 80)	<.001
Affected side				
1	Shoulder	0.99 (0.99-0.99)	455.606 (83, 83)	<.001
2	Shoulder	0.99 (0.99-0.99)	677.202 (83, 83)	<.001
2	Elbow	0.98 (0.98-0.99)	164.610 (83, 83)	<.001
3	Shoulder	0.99 (0.99-0.99)	1001.732 (83, 83)	<.001
3	Elbow	0.99 (0.98-0.99)	216.907 (83, 83)	<.001
4	Shoulder	0.99 (0.98-0.99)	260.500 (83, 83)	<.001
4	Elbow	0.99 (0.99-0.99)	959.581 (83, 83)	<.001

### Track-UL Algorithm Test-Retest Reliability for Laboratory Measurement

The Track-UL algorithm reliability results for both the shoulder and elbow joints on both sides are shown in Table 4. The ICC showed that the Track-UL algorithm had excellent reliability (all ICC values>0.81).

**Table 4.** Track-UL algorithm test-retest reliability for the laboratory measurements (N=27).

Tested side and task number	Joint	ICC^a^	SEM^b^	MDC^c^
Affected side				
2	Elbow	0.95	5.32	14.77
3	Elbow	0.91	11.78	32.65
4	Elbow	0.95	7.41	20.55
1	Shoulder	0.97	4.11	11.41
2	Shoulder	0.98	4.75	13.19
3	Shoulder	0.93	6.56	18.19
4	Shoulder	0.88	4.20	11.65
Unaffected side				
2	Elbow	0.84	3.83	10.62
3	Elbow	0.91	5.82	16.15
4	Elbow	0.93	2.90	8.04
1	Shoulder	0.92	5.19	14.39
2	Shoulder	0.89	7.20	19.96
3	Shoulder	0.87	5.43	15.07
4	Shoulder	0.97	1.87	5.20

a ICC: intraclass correlation coefficient.

b SEM: SE of measurement.

c MDC: minimum detectable change.

### Concurrent Validity for Home Measurement

**Figure 6.**
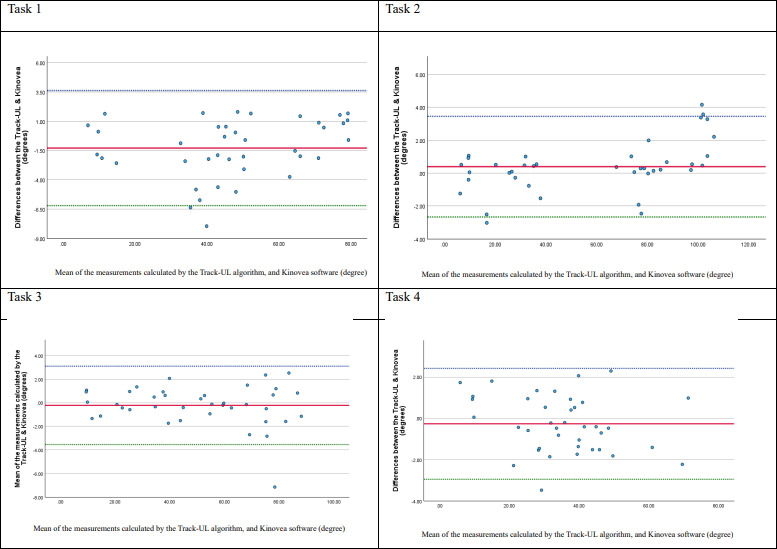
Bland-Altman plots for comparing the Track-UL algorithm and Kinovea software for the shoulder joint in the affected side in the home setting.

Only 48.1% (13/27) of the participants were able to record videos at home that were fit for analysis. For the acceptable videos, the Bland-Altman plots (Figures6  7) indicated that the mean difference in the shoulder and elbow joint angles between the Track-UL algorithm and Kinovea software was close to 0; the mean values ranged from −1.29 to 0.39 degrees, indicating no systematic bias. The 95% LoA ranged from −6.21 to 3.62 degrees for shoulder joint measurements and from −4.06 to 2.53 degrees for elbow joint measurements. This difference is not considered clinically significant [30] (Table 5).

**Figure 7.**
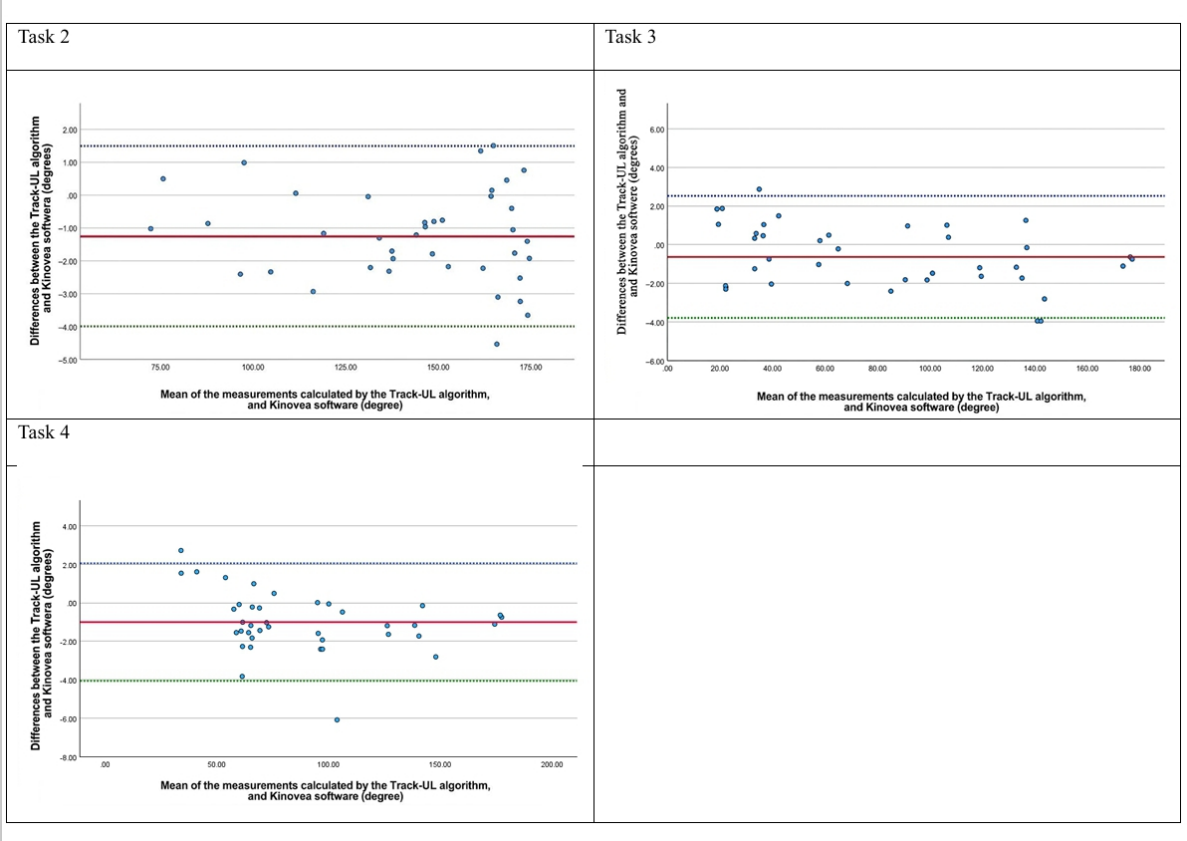
Bland-Altman plots for comparing the Track-UL algorithm and Kinovea software for the elbow joint in the affected side in the home setting.

There was no clear evidence of a trend or heteroscedasticity on the plots except for the shoulder joint during shoulder elevation (task 2), which displayed a proportional bias whereby the positive difference increased at higher angles of elevation and the negative difference increased at lower angles. However, the positive difference of up to 3.45 degrees and the negative difference of up to −2.66 degrees had no substantial clinical influence on measuring shoulder functional active ROM [30] (Table 5).

**Table 5.** The limit of agreement (LoA) between the Track-UL algorithm and the Kinovea software from videos recorded at home.

Tested side and task number	Elbow joint				Shoulder joint			
	Observations, n	Mean (SD; 95% CI)	Lower LoA	Upper LoA	Observations, n	Mean (SD; 95% CI)	Lower LoA	Upper LoA
Affected side								
1	—^a^	—	—	—	39	−1.29 (2.51; –2.11 to –0.47)	−6.21	3.62
2	39	−1.25 (1.39; –1.71 to –0.81)	−3.99	1.47	39	0.39 (1.56; –0.11 to 0.89)	−2.66	3.45
3	39	−.63 (1.61; –1.51 to –0.11)	−3.79	2.53	39	−0.22 (1.69; –0.77 to 0.32)	−3.54	3.09
4	39	−1.01 (1.56; –1.51 to –0.49)	−4.06	2.05	39	−0.27 (1.36; –0.71 to 0.17)	−2.95	2.41

a Not applicable.

### Absolute Agreement Analysis for Home Measurement

The absolute agreement between the 2 measurement tools (Track-UL algorithm and Kinovea software) for videos recorded at home was assessed using the ICC. The results demonstrated excellent reliability across all evaluated tasks and joints. ICC values ranged from 0.97 to 0.99, indicating a high degree of consistency between the 2 assessment tools (Table 6).

**Table 6.** Intraclass correlation between the Track-UL algorithm and the Kinovea software (home setting).

Side and task number	Joint	Intraclass correlation coefficient (95% CI)	*F* test (*df*)	*P* value
Affected side				
1	Shoulder	0.99 (0.97-0.99)	284.85 (38, 38)	<.001
2	Shoulder	0.99 (0.99-0.99)	2091.99 (38, 38)	<.001
2	Elbow	0.99 (0.98-0.99)	1772.51 (38, 38)	<.001
3	Shoulder	0.97 (0.95-0.98)	89.19 (38, 38)	<.001
3	Elbow	0.99 (0.98-0.99)	260.63 (38, 38)	<.001
4	Shoulder	0.99 (0.99-0.99)	467.71 (38, 38)	<.001
4	Elbow	0.99 (0.99-1.00)	2508.41 (38, 38)	<.001

### Track-UL Algorithm Test-Retest Reliability for Home Measurement

To assess the test-retest reliability of the Track-UL algorithm from the videos recorded at home, we collected 3 independent measures for each task. The ICC showed that the Track-UL algorithm had good to excellent reliability results for both the shoulder and elbow joints on the affected side (all ICC values≥0.778; Table 7).

**Table 7.** Track-UL algorithm test-retest reliability for the home measurement (n=13).

Tested side and task number	Joint	ICC^a^	SEM^b^	MDC^c^
Affected side				
2	Elbow	0.77	13.07	36.24
3	Elbow	0.98	5.94	16.48
4	Elbow	0.94	9.12	25.28
1	Shoulder	0.97	3.23	8.95
2	Shoulder	0.99	2.29	6.35
3	Shoulder	0.96	4.52	12.54
4	Shoulder	0.91	4.30	11.92

a ICC: intraclass correlation coefficient.

b SEM: SE of measurement.

c MDC: minimum detectable change.

## Discussion

### Principal Findings

The main aims of this study were to measure the concurrent validity, absolute agreement, and test-retest reliability of the newly developed Track-UL algorithm. This algorithm uses a 2D markerless motion capture system based on MediaPipe, a pose estimation framework, to measure shoulder and elbow functional active ROM from recorded videos in people with stroke. While most algorithms that use MediaPipe pose estimation models have been tested and implemented in healthy populations for a variety of applications [31-33], such as assessment for spinal diseases and frozen shoulder [34], among people with tremors [35], and for telerehabilitation purposes [36,37], they have not yet been validated in individuals with stroke using complex functional tasks. The use of computer vision–based assessment of functional movement in people with stroke has the potential to improve the accessibility and scalability of movement assessment by providing accurate, low-cost, and markerless measurement outside of specialist laboratory settings. Therefore, we assessed shoulder and elbow functional active ROM during 4 complex functional tasks in both laboratory and home settings to explore the efficacy and effectiveness of this approach for individuals with stroke. We also present the possibilities of using this technique for telemonitoring in stroke rehabilitation.

For the videos recorded by the researcher in the laboratory, we observed a positive agreement between the Track-UL algorithm and the Kinovea software in the measurement of upper-limb function in survivors of stroke in 4 different tasks. The LoA was below the minimum clinically significant difference, which supports the use of the Track-UL algorithm in clinical practice to evaluate and monitor the progression of upper-limb kinematics. The LoA was slightly higher in certain movements on the affected side relative to the unaffected side, potentially due to the compensatory shoulder joint movements (internal rotation during arm elevation) and elbow joint movements (flexion and extension), which influenced the process of analysis.

We observed a significant degree of absolute agreement between the Track-UL algorithm and the Kinovea software in videos captured by both the physiotherapist and the participants. This finding supports the interchangeability of tools as the Track-UL algorithm performs as an intuitive and convenient tool for assessing arm mobility in both clinical and remote settings. We support the integration of this tool into the clinical assessment of upper-limb kinematics in individuals with stroke. This tool offers significant advantages, particularly for those who experience difficulties commuting to clinics or who reside in rural areas, by enabling remote follow-up and monitoring of upper-limb recovery. Furthermore, it provides more convenient solutions for carers, reducing their time and financial burdens as a result of traveling to clinics with their relatives with stroke. We can integrate this tool with any telerehabilitation model to provide a telerehabilitation and telemonitoring tool for people to use at home.

The test-retest reliability of the Track-UL algorithm demonstrated excellent consistency and robustness in repeated clinical assessments both in laboratory and home environments. The findings indicate that the Track-UL algorithm can deliver highly reliable kinematic measurements for recorded videos of people with stroke, rendering it appropriate for both in-clinic and remote assessments in stroke rehabilitation. This can be clinically beneficial, particularly for individuals reporting fatigue, allowing us to request that they perform the task only once rather than multiple times.

We calculated the minimum detectable change of the Track-UL assessment tool and determined that it was slightly elevated for the elbow joint compared to the shoulder joint, more pronounced on the affected side. This is potentially attributable to personal variability and the complexity of the tasks, which emphasize functional movements rather than simple joint movements. Additionally, the use of a 2D markerless motion capture system limits measurements to a single plane, making the results more sensitive to compensatory movements such as whole-arm rotation. Therefore, if this algorithm is to be used for monitoring upper-limb kinematics over time as a telemonitoring tool, the data should be interpreted with caution in clinical practice.

Our findings align with those of Latreche et al [31], who reported good agreement and reliability of an algorithm using the MediaPipe pose estimation framework in comparison to a goniometer; however, their study focused on simple shoulder movements. In our study, we explored complex functional arm movements involving the shoulder and elbow joints in people with stroke, which were close to what we usually evaluate in clinical practice. Another study measured upper-limb kinematics using sensorized gloves equipped with electromagnetic sensors capable of assessing 6 df during functional reach and grasp tasks [38]; however, the Track-UL algorithm–based measurement tool has the potential to be more user-friendly, requiring no additional equipment, which increases its suitability for clinical use.

Many participants were either unable to accurately record videos at home (4/27, 14.8%), potentially because restricted home environments, such as small houses, made it difficult to achieve the required camera angle, or they were unable to record the videos at all (10/27, 37%) due to technical difficulties in following the video recording instructions and then sending the videos back to the research team, particularly among older survivors. Consequently, we could not analyze the videos to determine whether the setup was incorrect, whether the entire movement was captured within the camera’s frame, or whether the camera was positioned arbitrarily, all of which could affect pose estimation analysis. All of these factors affected the ability to record the videos at home and the ability of the algorithm to analyze the joint angles from the videos recorded independently by the participants or their carers at home. We recommend integrating this algorithm into an application that can offer a step-by-step guide for video recording alongside real-time feedback on the video quality, such as the camera angle, the distance from the camera, and frame coverage, to enhance the feasibility of using this algorithm as a telemonitoring tool for people with stroke.

For the current application, which aimed to validate 4 functional active ROM tasks, 2D analysis is appropriate. However, there are known limitations, such as lack of depth information and issues with out-of-plane movement detection. These could potentially be addressed using 3D with 2D recorded videos to yield further depth of information that could be useful in other contexts. We also recommend incorporating more functional tasks into the assessment and cross-validating them with functional questionnaires (FMA-UE). In addition, time-series analysis could yield extra layers of useful clinical information, including qualitative considerations such as movement smoothness, which could add further insight in future studies.

### Conclusions

The Track-UL algorithm can be adopted as a cost-effective, fast, and user-friendly alternative to traditional motion analysis software such as Kinovea. This allows for its use in both clinical and potentially in remote settings. This technology has the potential to benefit people with stroke through monitoring and motor performance assessments. We recommend developing an application with an easy-to-use interface that enables survivors of stroke to securely record and analyze their arm movement both in clinics and at home. Automated reports can provide physiotherapists with objective, measurable kinematic data to help design and adjust personalized rehabilitation programs.

## Supplementary material

10.2196/87128 Multimedia Appendix 1 Instructions for recording the videos.

## Abbreviations

FMA-UE: Fugl-Meyer Assessment for the upper extremity
ICC: intraclass correlation coefficient
LoA: limit of agreement
ROM: range of motion
