# Supplementary material for: Validity and Reliability of the Track-UL Algorithm Compared With Kinovea Software for Measuring Upper-Limb Functional Range of Motion in People After Stroke: Cross-Sectional Observational Study
Source: JMIR Rehabil Assist Technol. 2026 May 11;13:e87128. doi: 10.2196/87128 (PMC13160480; doi:10.2196/87128)
Supplement: Multimedia Appendix 1 [file rehab-v13-e87128-s001.pdf]

Instructions for recording the videos.

All the tasks will be done from the setting position (armless chair), and the chair should be close to the wall. You will have to measure the distance between the chair legs as illustrated in the following photos.

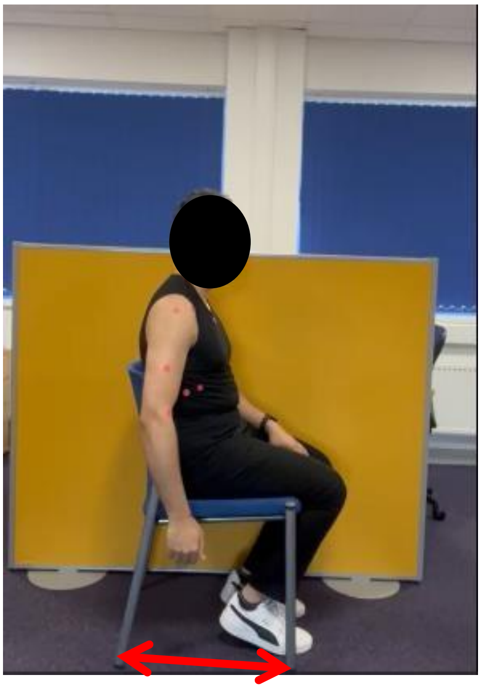

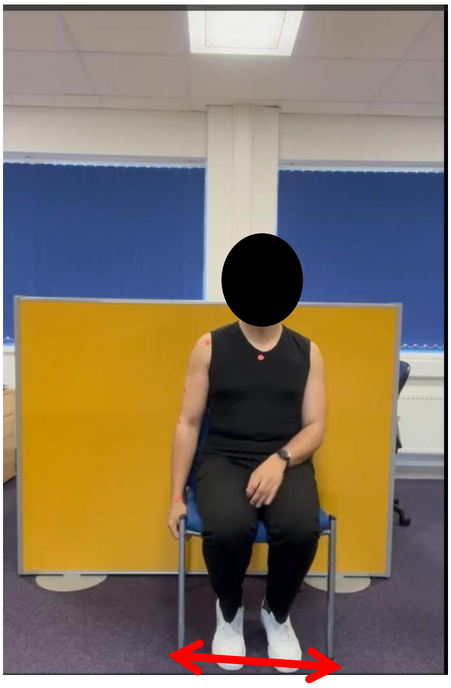

The camera should be 1.5 meters away from the chair.

The camera should be able to see all of your upper, and lower body, and the legs of the chair.

For each task, there will be instructions regarding the starting position.

Task 1: Arm abduction from Frontal view. (Affected arm)

Camera position: in front of the chair (please see the picture below).

Starting position: sitting on a chair with back fully rested on the chair. The arm is at the side of the body.

Task: try to move your arm with a straight elbow away from your body as much as you can without moving your trunk, then back again to the starting position.

Repeat this movement 3 times.

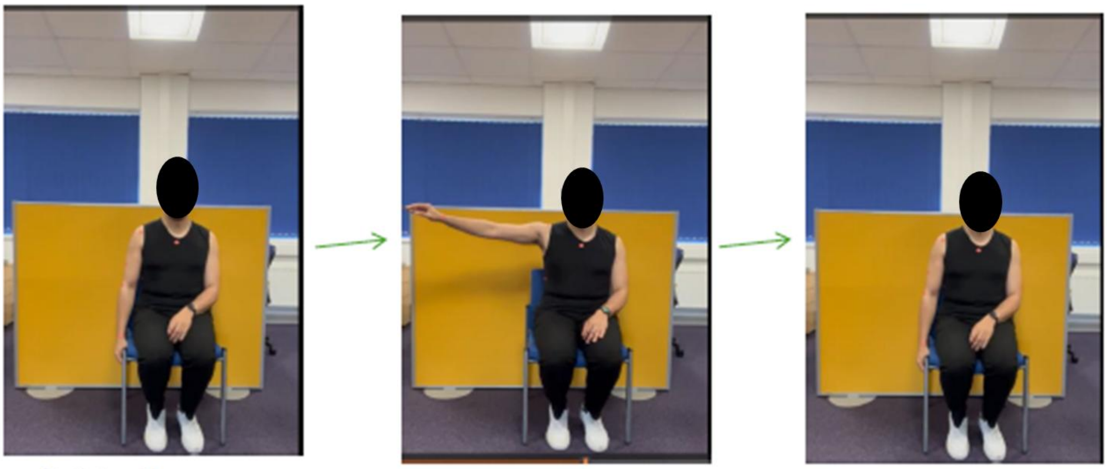

Starting position

End position

Task 2: Arm elevation from Sagittal view. (Affected arm)

Camera position: Lateral to the chair (please see the picture below).

Starting position: sitting on a chair with back fully rested on the chair. The arm is at the side of the body.

Task: try to elevate your arm with a straight elbow in front of your body as much as you can without moving your trunk, then back again to the starting position.

Repeat this movement 3 times.

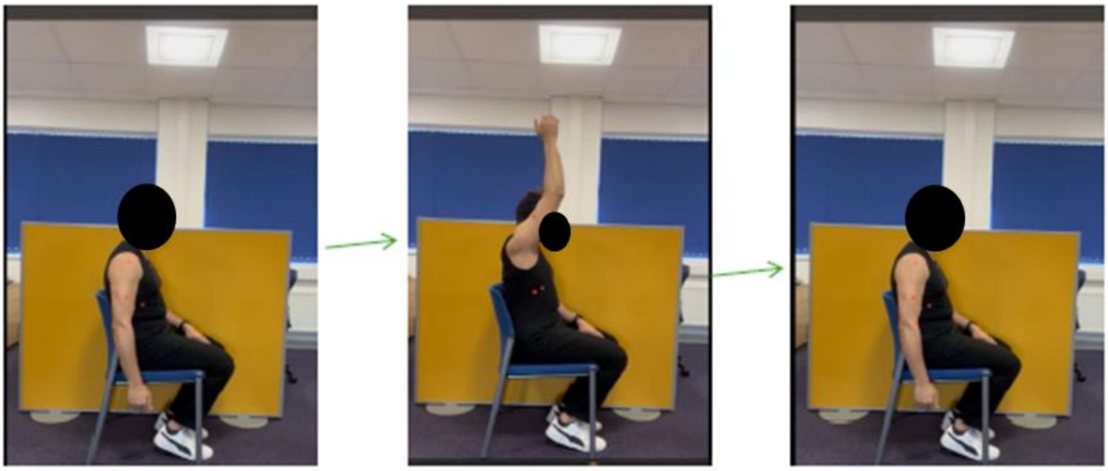

Starting position *End position*

Task 3: Hand to head from Sagittal view. (Affected arm)

Camera position: Lateral to the chair (please see the picture below).

Starting position: sitting on a chair with back fully rested on the chair. The hand is resting on the leg.

Task: try to move your hand and bring it over your head without moving your trunk, then back again to the starting position.
Repeat this movement 3 times.

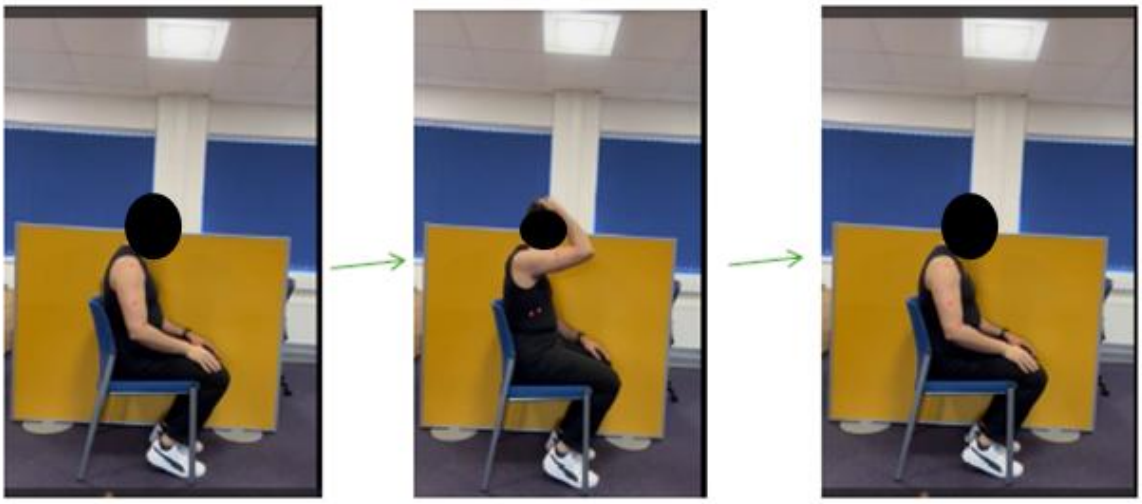

Starting position End position

Task 4: Hand to mouth from Sagittal view. (Affected arm)

Camera position: Lateral to the chair (please see the picture below).

Starting position: sitting on a chair with back fully rested on the chair. The hand is resting on the leg.

Task: try to move your hand and bring it over your mouth without moving your trunk, then back again to the starting position.

Repeat this movement 3 times.

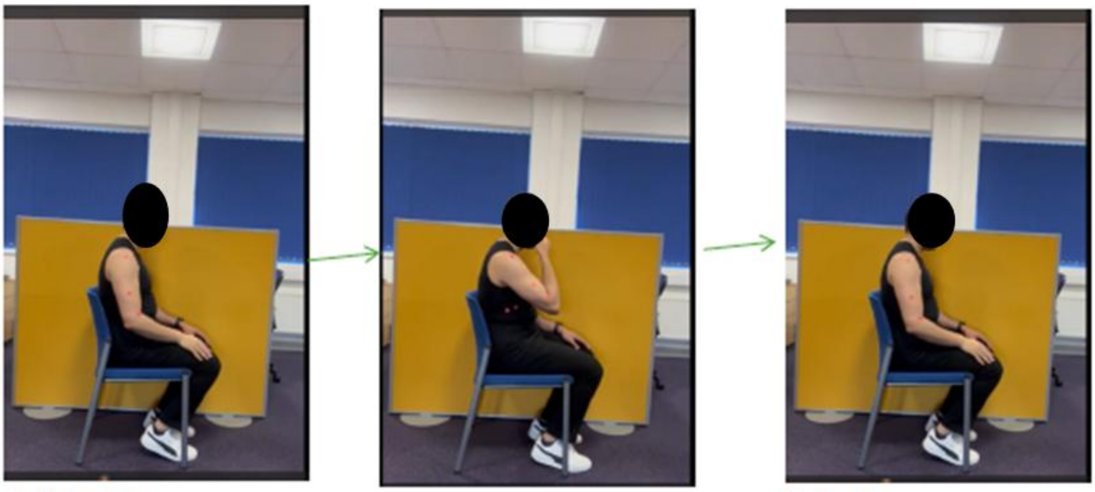

Starting position *End position*
